# LETTER TO THE EDITOR Correction of Tethered Tracheostomy Scar Using Dermofat Graft

**Published:** 2012-11-07

**Authors:** Fikret Eren, Cenk Melikoglu, Deniz Kok, Salim Iskender

**Affiliations:** ^a^Department of Plastic and Reconstructive Surgery, Etimesgut Military Hospital, Ankara; ^b^Department of Plastic and Reconstructive Surgery, Sanliurfa Training and Research Hospital, Sanliurfa, Turkey

Dear Sir,

We present a 43-year-old man with ca larynx who underwent tracheostomy, secondary to subtotal laryngectomy operation. The patient's respiratory problems were resolved and tracheostomy was removed. He presented to our clinic with a depressed scar and a tracheal tug (Fig 1).

Tracheostomy is a life-saving maneuver used in respiratory emergencies, maxillofacial trauma, and oncologic surgery with extensive resection. After decannulation, final complication is a depressed scar with up-and-down movement during swallowing. As secondary healing of a tracheostomy proceeds some adhesions occurred between trachea and skin creating a tracheal tug as the patient swallows.^1^ Rosenbower et al^2^ found 10% rate of dysphagia and poor scaring postoperatively.

The patient was administered a local anesthesia. Incision was planned horizontally so that it passed in the midline of the tracheostomy scar. Adherent skin was dissected from the trachea and dissection was extended approximately 2 cm inferiorly and superiorly. If a fistula remained at the trachea, a small cuff of tissue might have been left attached to the trachea.^3^ Dermofat graft taken from right groin was placed as an interpositional graft into the pouch to correct and augment the depressed scar and prevent tracheal adhesions to the adjacent skin. Incisions were repaired with 5/0 Prolene. The sutures were removed on the seventh day following surgery. No postoperative problems were encountered (Fig 2). The follow-up period was 36 months, and the long-term results of the treatment were satisfactory (video).

Poulard,^4^ in 1918, described filling the defect with deepithelialized scar and reapproximating the skin flaps. The literature describes several management modalities involving repairing the defect with placing a tube-shaped scar into the defect,^5^ mobilization of sternal heads of the SCM into the scar,^6^ Z plasty,^7^ advancement of platysma,^8^ and revision by using an allograft.^9^ However, fibrosis and synechia in the surgical area make these techniques impractical and difficult to perform.

We conclude that repairing the tethered tracheostomy scars with dermofat graft is a simple and reliable method with better short- and long-term aesthetic and functional outcomes.

## Figures and Tables

**Figure 1:**
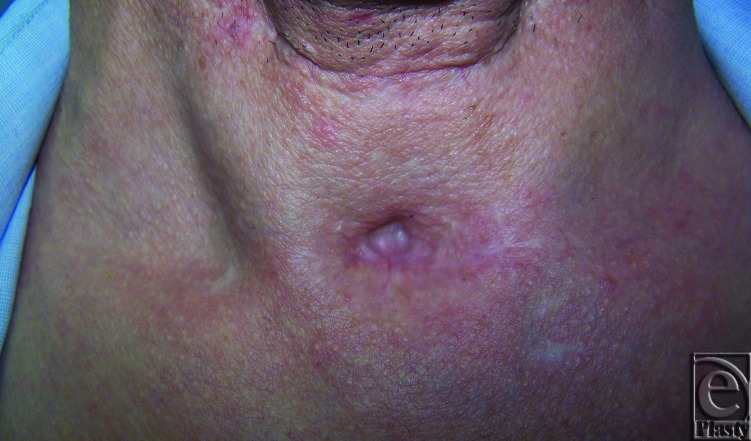
Preoperative view.

**Figure 2:**
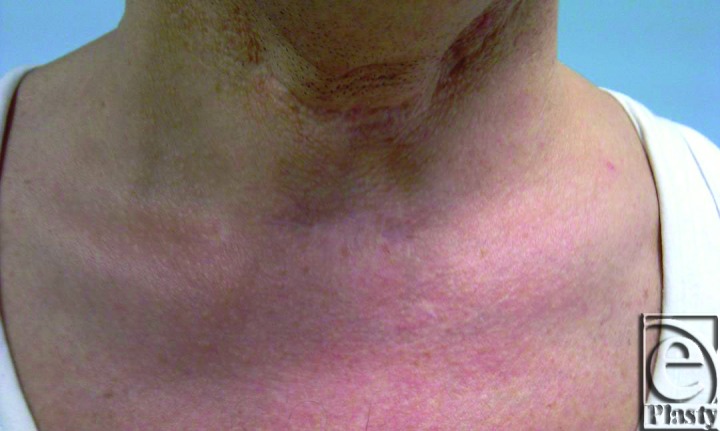
Thirty-six-month postoperative result.

**Video:.** Thirty-six-month postoperative result during swallowing (video).
